# Rodent Activity Detector (RAD), an Open Source Device for Measuring Activity in Rodent Home Cages

**DOI:** 10.1523/ENEURO.0160-19.2019

**Published:** 2019-07-09

**Authors:** Bridget A. Matikainen-Ankney, Marcial Garmendia-Cedillos, Mohamed Ali, Jonathan Krynitsky, Ghadi Salem, Nanami L. Miyazaki, Tom Pohida, Alexxai V. Kravitz

**Affiliations:** 1 National Institute of Diabetes and Digestive and Kidney Diseases, National Institutes of Health, Bethesda, MD 20892; 2 Signal Processing and Instrumentation Section, Office of Intramural Research, Center for Information Technology (CIT), National Institutes of Health, Bethesda, MD 20814; 3 National Institute on Drug Abuse, National Institutes of Health, Baltimore, MD 21224

**Keywords:** continuous activity monitoring, home cage, motion detector, physical activity

## Abstract

Physical activity is a critical behavioral variable in many research studies and is, therefore, important to quantify. However, existing methods for measuring physical activity have limitations which include high expense, specialized caging or equipment, and high computational overhead. To address these limitations, we present an open-source, cost-effective, device for measuring rodent activity. Our device is battery powered and designed to be placed in vivarium home cages to enable high-throughput, long-term operation with minimal investigator intervention. The primary aim of this study was to assess the feasibility of using passive infrared (PIR) sensors and microcontroller-based dataloggers in a rodent home cages to collect physical activity records. To this end, we developed an open-source PIR based data-logging device called the rodent activity detector (RAD). We publish the design files and code so others can readily build the RAD in their own labs. To demonstrate its utility, we used the RAD to collect physical activity data from 40 individually housed mice for up to 10 weeks. This dataset demonstrates the ability of the RAD to (1) operate in a high-throughput installation, (2) detect high-fat diet (HFD)-induced changes in physical activity, and (3) quantify circadian rhythms in individual animals. We further validated the data output of the RAD with simultaneous video tracking of mice in multiple caging configurations, to determine the features of physical activity that it detects. The RAD is easy to build, economical, and fits in vivarium caging. The scalability of such devices will enable high-throughput studies of physical activity in research studies.

## Visual Abstract

**Figure:**
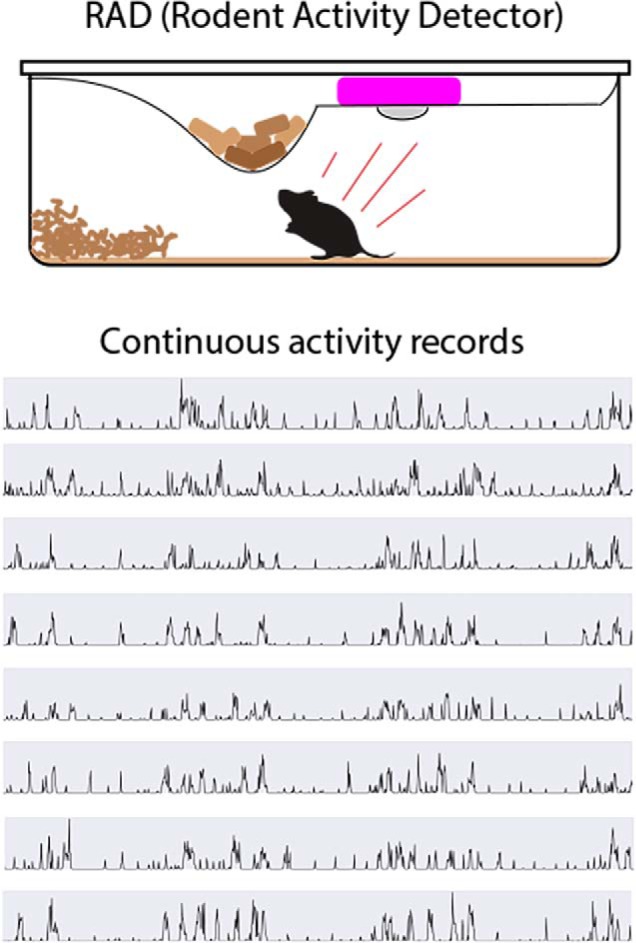

## Significance Statement

Physical activity is an important determinant of human health, which reduces the risk of cardiovascular, metabolic and neurologic disorders. Better methods for tracking physical activity in research studies may lead to insights that improve physical activity levels in people. To this end, we designed and built an inexpensive device to continuously track home-cage activity levels in rodents. Our device, rodent activity detector (RAD), allows for high-throughput activity monitoring with minimal investigator intervention, presenting an economical and efficient option for investigating physical activity levels in research studies.

## Introduction

Physical activity is an important component of healthy lifestyles and has been linked to reductions in the risk of several major disorders including type 2 diabetes, obesity, and cardiovascular disease (Michigan et al., 2011; Roemmich et al., 2014; Silverman and Deuster, 2014; Benatti and Pedersen, 2015). Despite this link between physical activity and health, relatively little is known about how physical activity exerts its beneficial effects. Several fundamental questions remain unanswered: how much activity is needed to reduce the risk of these diseases? Do different individuals require different amounts of physical activity to experience health benefits? And how does physical activity modulate complex disease states at the circuit or molecular levels?

Rodents provide an excellent model system for gaining insight into these questions (Novak et al., 2012). However, accurately measuring physical activity levels in rodents remains a challenge. Commonly used methods fall broadly into two types: (1) specialized arenas, which track mouse activity with infrared “beam breaks” or video cameras, and (2) home-cage devices that count wheel rotations or sensor activations. The first approach is often low-throughput, as it requires expensive equipment and dedicated laboratory space. Due to these limitations, activity is often measured in short daily sessions, sampling a fraction of daily activity, often in a novel environment. The second class of home-cage methods allow for continuous full-day monitoring, using either wheels or sensors. The presence of a running wheel is not always ideal as the wheel itself alters activity patterns and induces mice to move more (O'Neal et al., 2017), which may confound other factors that affect activity (Copes et al., 2015; de Carvalho et al., 2016; O'Neal et al., 2017). Other groups have used passive infrared (PIR) sensors (Tamborini et al., 1989; Brown et al. (2016), capacitive sensors (Giles et al., 2018; Iannello, 2019; Pernold et al., 2019), and microwave based activity monitors (Pasquali et al., 2006; Genewsky et al., 2017) to measure home-cage activity. Here, we aimed to complement and enhance these existing methods by designing an easy to build, cost-effective, and open-source device. An ideal solution for measuring physical activity would be home-cage compatible, collect activity data continuously with no human intervention, and be scalable to large installations of cages. In line with these requirements, we present a simple PIR-based activity logger, the rodent activity detector (RAD). The low cost and simple design of the RAD renders it ideal for activity monitoring in high-throughput experiments and multi-site studies. We make our device design files and code freely available to the research community to build, use, and improve.

## Materials and Methods

### Animals

A total of 47 adult male mice (C57Bl/6 background) were singly housed in murine vivarium caging (Allentown NextGen caging) in a 12/12 h light/dark cycle at room temperature. Mice were given *ad libitum* access to standard chow (5001 Rodents Diet; LabDiet) and water unless otherwise noted, and cages were changed every two weeks. All animal procedures were performed in accordance with the National Institute of Diabetes and Digestive and Kidney Diseases/National Institutes of Health animal care committee’s regulations.

### Device design

**Figure 1.**
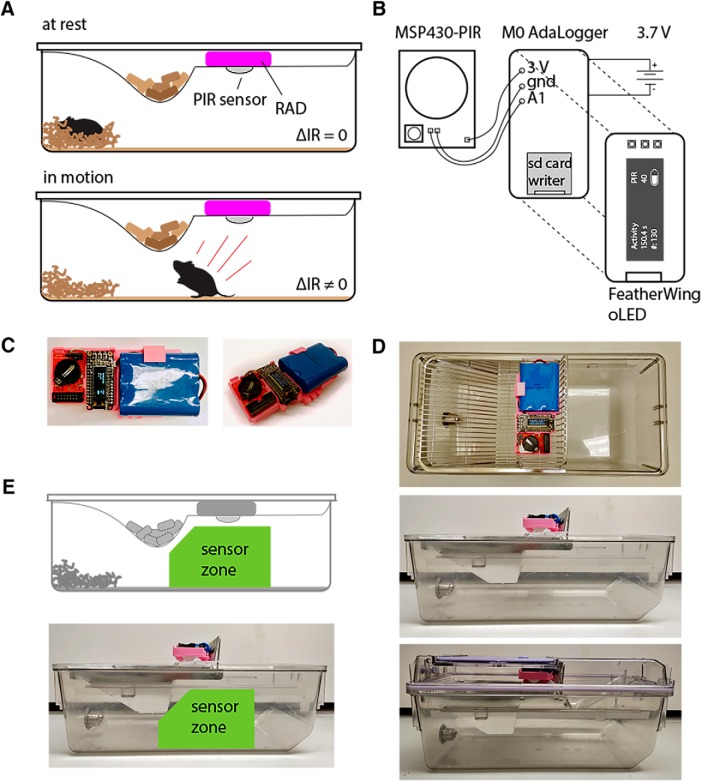
RADs for high-throughput longitudinal recording of rodent physical activity. ***A***, Schematic showing location of assembled RAD in rodent home cage. PIR sensor is activated by animal locomotion. ***B***, Diagram of PIR sensor (MSP430-PIR, Olimex), SD logger (M0 AdaLogger, Adafruit #2796), and organic LED shield (FeatherWing, AdaFruit #2900) connected to a 3.7-V lithium ion battery (Adafruit #353). Components are soldered and (***C***) housed in a 3D-printed casing (for Arduino code and 3D printing files, see https://hackaday.io/project/160742homecage-activity-monitoring-with-pirs) and fitted on top of the chow tray, which is then placed in the cage (***D***). ***D***, Top and side views of RAD fitted onto the chow try and placed in the home cage. ***E***, Green “sensor zone” showing where RAD monitors activity in the home cage.

Our method relies on small, low-cost, PIR sensors, which are battery powered and run for weeks in a home-cage setting. The device cost is significantly less expensive than commercial solutions, costing ∼$85 to build each device (Table 1). These devices can be built with minimal electronics or coding experience, fit in traditional vivarium caging (Fig. 1) allowing for high throughput experiments without requiring additional laboratory space, and track home-cage activity levels without the use of a running wheel. PIR sensors have several inherent characteristics that make them ideal for this application. They are very low power, capture data at a low rate that facilitates long-term collection, and they do not detect movement outside of the plastic walls of the cage, so they are not triggered by movement in other cages.

**Table 1.** Specifications

Hardware name	RAD
Subject area	Behavioral neuroscience
Hardware type	In-lab sensor
Open source license	GNU General Public License v3.0
Cost of hardware	$85
Source file repository	https://hackaday.io/project/160742homecage-activity-monitoring-with-pirs

RAD comprises several inexpensive components (Table 2). We modified a PIR sensor (MSP430-PIR, Olimex) to connect the output to a digital input on a microprocessor equipped with a microSD card logger (M0 AdaLogger, Adafruit #2796) and oLED screen for displaying data (FeatherWing, AdaFruit #2900; Fig. 1*B–D*; Arduino code for the device is included as a supplement and available online at https://hackaday.io/project/160742homecage-activity-monitoring-with-pirs). We chose this PIR sensor for its low power consumption, but many other PIR sensors will work as drop in replacements in our design. We powered the device with a 6600 mAh battery (Adafruit #353), which was calculated to last 18 d, and empirically tested to last at least 14 d. If sleep modes are used and optimized on the microprocessor, we estimate the battery life could be extended to several weeks or months. Additionally, the RAD can be wall powered with a microUSB cable for uninterrupted use, if the caging is compatible with such a cable. We printed and assembled 40 RAD devices, and mounted them in rodent home cages for continuous, high-throughput activity monitoring (Fig. 1*D*).

**Table 2.** Bill of materials

Component	Cost per unit	Source of materials
PIR sensor	$14.53	Olimex, product #MSP430-PIR
Jumper wires	$1.95	Adafruit, product #1956
M0 Adalogger	$19.95	Adafruit, product #2796
FeatherWing oLED	$14.95	Adafruit, product #2900
Battery	$29.50	Adafruit, product #353
SD card	$5.39	NewEgg, product #9SIAC3J63S1330
3D printed housing	–	–
Total	$86.27	

All components needed to build one RAD device.

### Build instructions

RAD device fabrication, assembly, and programming are outlined at https://hackaday.io/project/160742homecage-activity-monitoring-with-pirs. All design files necessary to complete this build (including electronic layout/soldering instructions, Arduino code, and 3D printing design files) are included as supplementary files and also located at https://hackaday.io/project/160742homecage-activity-monitoring-with-pirs. In brief, download and install Arduino IDE (https://www.arduino.cc/en/Main/Software), as well as support for the Adalogger board (https://learn.adafruit.com/adafruit-feather-m0-adalogger?view=all). Install the relevant libraries (Adafruit_SSD1306.h, Wire.h, RTCZero.h, SPI.h, SdFat.h, Adafruit_GFX.h). Download the PIR device sketch (PIRCounter-091218.zip, https://hackaday.io/project/160742homecage-activity-monitoring-with-pirs). Download and print the PIR housing 3D STL file (PIR monitor housing, https://hackaday.io/project/160742homecage-activity-monitoring-with-pirs). Install male headers on the oLED shield and female headers on the Adalogger M0 board. Solder wires to the PIR sensor signal, ground, and 3V power connections. Solder these wires to the corresponding power and ground connections on the oLED shield, and signal wire to the A1 input on the Adalogger (Fig. 1*B*). Plug the oLED into the Adalogger, and attach the battery. Flash the RTC setting code and the PIR counter device code to the Adalogger (https://hackaday.io/project/160742homecage-activity-monitoring-with-pirs). Assemble the RAD electronics into the RAD housing (Fig. 1*C*). Secure the RAD device on top of the food hopper. This specific device housing was designed to be compatible with Allentown NextGen wire racks but can be modified for compatibility with other caging.

### Method of sensing

PIR sensors detect when warm moving objects (such as an animal) cross its sensing zone. Importantly, PIR sensors do not detect activity through glass or plastic, making them an ideal sensor for this application as they are not triggered by activity in neighboring cages. By mapping the area of activation using an infrared LED, we determined that in Allentown NextGen wire rack home cages with RAD placed above the wire rack, RAD tracks activity in ∼30% of the cage surface area (Fig. 1*E*).

### Operation instructions

To start logging activity data, press the “A” button on the oLED shield. To update the time and date, press the “B” button. Once started, RAD will count the number of PIR “active bouts,” as well as the total duration the sensor is active each minute. The RAD ends each “activity bout” after 2 s with no activity detected from the sensor. The total active duration, as well as the number of activity bouts, are recorded on an internal microSD card at a frequency of once per minute. These variables and logging frequency can be modified in the Arduino sketch.

### Data analysis

RAD logs data in CSV files stored on a microSD card (Table 3). Data are logged once per minute and include a timestamp, time-elapsed timestamp, device name, a cumulative measure of how many active bouts were recorded per minute (PIRCount), a cumulative measure of the duration of those active bouts per minute (PIRDuration), and battery voltage. Example data shown in Table 3. At the end of the study, the SD card can be removed and the CSV files can be opened to view the data. We also provide Python analysis scripts that may be helpful for visualizing data (available as a supplement and at https://hackaday.io/project/160742homecage-activity-monitoring-with-pirs). These scripts are not necessary, as any graphing program can be used to visualize and analyze the data.

**Table 3.** Example of RAD data logged in a csv file

MM:DD:YYYY hh:mm:ss	Elapsed time	Device	PIRCount	PIRDuration	BatteryVoltage
10/25/2018 11:10	0:01:00	39	15	21.51	4.18
10/25/2018 11:11	0:02:00	39	19	26	4.17
10/25/2018 11:12	0:03:00	39	27	32.43	4.15
10/25/2018 11:13	0:04:00	39	40	46.9	4.16
10/25/2018 11:14	0:05:00	39	49	53.4	4.16
10/25/2018 11:15	0:06:00	39	55	59.18	4.16
10/25/2018 11:16	0:07:00	39	68	70.78	4.16
10/25/2018 11:17	0:08:00	39	77	78.87	4.17

### Statistical analysis

Statistical tests were performed using the StatsModels python module. Two-way ANOVAs were used to compare group means over time (Fig. 4); *p* > 0.05 was considered statistically significant.

### Code accessibility

The code/software described in the paper is freely available online at https://hackaday.io/project/160742homecage-activity-monitoring-with-pirs. The code is available as Extended Data 1.

10.1523/ENEURO.0160-19.2019.ed1 Extended Data 1 Data contain the following files: “AdaLoggerM0-SetClock” contains code for setting the real time clock on the Adalogger M0 board; “RAD_activity_counter” contains the main Arduino code to run RAD; “PIR monitor housing” is a 3D file of the housing; “RAD Sample Data” contains sample data from four devices over multiple days; “RAD_analysis” contains python scripts for data anlaysis; “RAD_libraries_031219” contains the Arduino libraries required for RAD. Download Extended Data 1, ZIP file.

## Results

### Device validation with video monitoring

**Figure 2.**
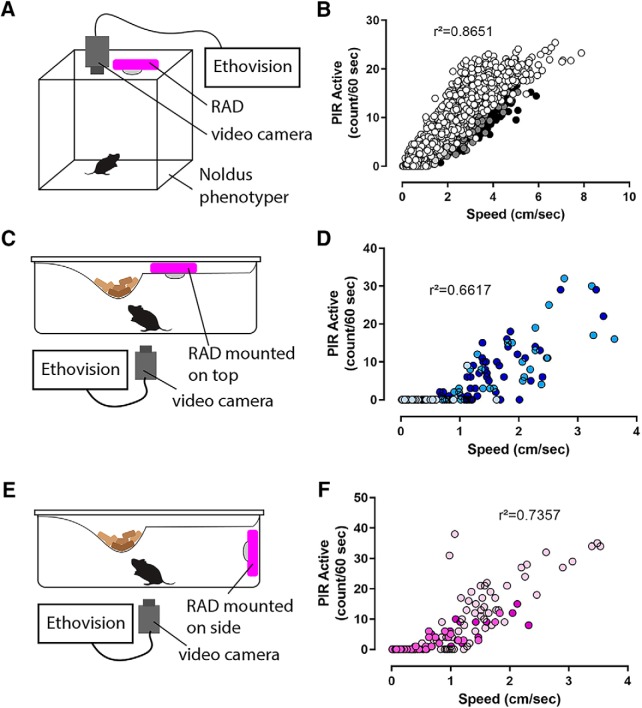
Data recorded by RAD correlates with speed. ***A***, Diagram showing setup of 24-h video-tracking PIR validation experiment in Noldus phenotyper. ***B***, Scatter plot of PIR sensor binned data versus video-tracked distance moved for bins of 60 s showing strong correlation between PIR sensor binned data and tracked mouse speed; *R* ^2^ = 0.8651, *N* = 4 mice, data from each mouse are shaded separately. Diagrams showing setups of home cage video-tracking validation for top (***C***) and side (***E***) RAD locations. Scatterplots show correlations between PIR active bouts per minute and distance moved per minute for top-setup (***D***) and side-setup (***F***); *R* ^2^ = 0.6617 and *R* ^2^ = 0.7357 for top and side setup, respectively; *N* = 3 mice, data from each mouse are shaded separately.

To quantify how the RAD output correlated with rodent activity, we recorded simultaneous video and PIR sensor data from four mice using an overhead camera over 24 h (Noldus Phenotyper; Fig. 2*A*). We processed the video with a commercial motion tracking software (Ethovision XT13, Noldus), and calculated velocity in 1-min bins (Fig. 2*B*). Coefficient of determination (*R* ^2^) was calculated for a linear regression between speed in each time bin and length of time the PIR was active in that same bin. The PIR data correlated strongly with speed (*R* ^2^ = 0.87, Fig. 2*B*). We explored changing the bin-width parameter and found that the coefficient of determination dropped off with shorter bin widths (30 s: *R* ^2^ = 0.84, 10 s: *R* ^2^ = 0.67, 1 s: *R* ^2^ = 0.33). Thus, we conclude that the PIR sensor in the RAD is useful for quantifying changes in activity that occur across minutes, but not second-by-second changes. Based on this validation result we set the default data logging frequency of the RAD to once per minute, but this parameter can be adjusted in the code.

To test whether RAD output correlates well with locomotion when used in a smaller home-cage setting, we recorded movement by positioning a video camera below the transparent plastic home cage (Allentown), and placing RAD either above the home-cage food hopper (Fig. 2*C*) or to the side of the cage wall (Fig. 2*E*). Recordings were conducted for 45–60 min and data were binned into 1-min bins (*n* = 3 mice). RAD activity correlated well with velocity moved for both top-mounted (*R* ^2^ = 0.66; Fig. 2*D*) and side-mounted (*R* ^2^ = 0.74; Fig 2*F*) configurations. For both RAD locations, these correlations reveal that activity was only detected by RAD when the mouse was in locomotion above 1 cm/sec. It is important to note that the sensing mechanism of RAD (PIR sensing) requires “line of sight” to the mouse, so for side-mounting RAD must be placed in the cage itself, or the caging modified if attached to the outside. If placed in the cage, the device should be protected with an enclosed housing or barrier to avoid mice chewing on the exposed electronics. To explicitly evaluate which behaviors the RAD detects, we used a behavioral classifier (Ethovision, Noldus) to score the Phenotyper videos for walking, resting, grooming, rearing, sniffing, and digging. In this analysis, “walking” correlated well with how long the PIR was active (*R* ^2^ = 0.61), while *R* ^2^ for all other behaviors were poorly correlated (*R* ^2^ < 0.2). We conclude that the PIR detects locomotion from place to place, but not slow speed or “in place” actions such as grooming and digging.

### High-throughput longitudinal monitoring of rodent physical activity in home cages

**Figure 3.**
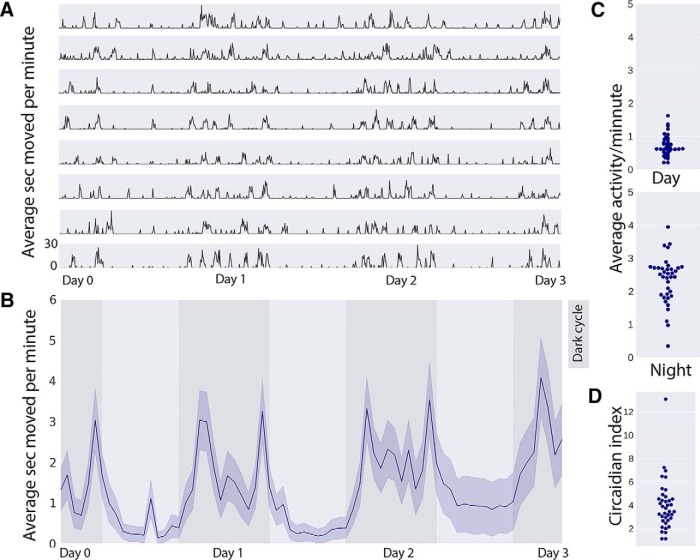
RAD records continuous activity. Traces show average activity over time (active seconds per minute) for eight individual mice (***A***) and pooled from *n* = 40 mice (***B***, ***C***) for 3 d (***B***). Scatterplots (***C***) showing average activity during the day (light cycle) and night (dark cycle) for 40 mice, as well as calculated circadian indices (***D***), illustrate heterogeneity across the sample population. Data in ***B*** are presented in mean activity (dark line) ± SEM (shaded lines).

To evaluate the use of the RAD in a high-throughput application, we built and positioned 40 RADs in mouse home cages for 8 d of data collection (Fig. 3). We observed the expected circadian rhythms in the activity records, wherein the RAD motion detection increased during the dark cycle and dropped during the light cycle (Fig. 3*A*-*C*). We also calculated a circadian index for each mouse and noted a large amount of heterogeneity among mice (Fig. 3*D*). We conclude that the PIR device can detect individual differences among mice and may be useful for studying circadian biology, with the caveat that behavioral detection is limited to periods of active wake and locomotion.

### RAD detects high-fat diet (HFD)-induced decreases in activity

**Figure 4.**
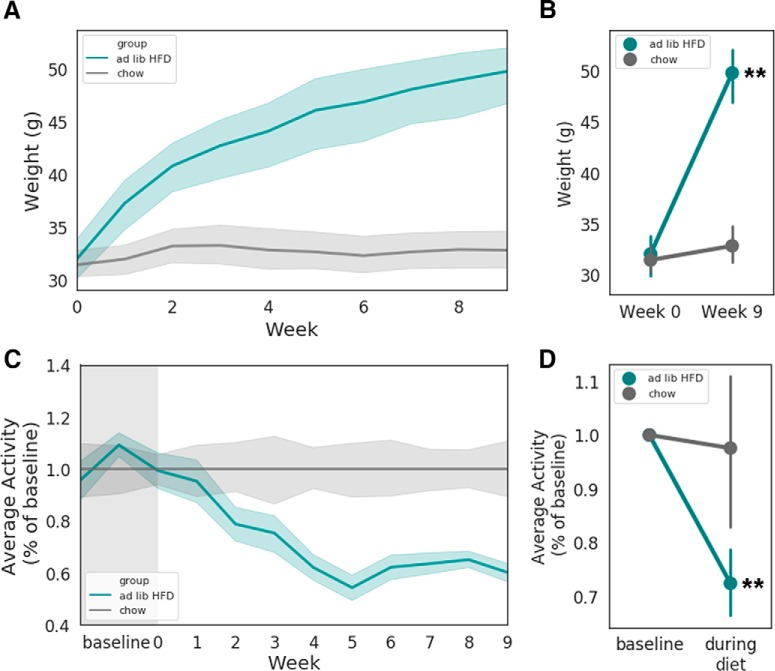
*Ad libitum* HFD decreases mouse home cage activity over time. ***A***, Time course of mouse weight over time. ***B***, Point plot showing average weights at weeks 0 and 9; HFD group had significant increase in weight compared to chow; *p* = 3.475723e-09, *F* = 60.093562. ***C***, Time course showing, over nine weeks, mice fed HFD exhibited decreasing activity over time relative to chow controls. ***D***, Point plot showing average activity levels normalized to baseline between chow and HFD groups; HFD group had significantly decreased average activity levels compared to chow; *p* = 0.000997, *F* = 13.300153. In ***B***, ***D***, two-way ANOVAs performed, stats reported for significant interactions between group and time; *n* = 10 mice per group.

To ask whether the data collected using RAD can reveal phenotypic differences between pathologic states, we tracked the activity data of 20 mice fed different diets. Ten mice were given *ad libitum* HFD, which leads to an increase in body weight and a decrease in activity levels (Friend et al., 2017). Over nine weeks of HFD exposure, mice gained weight relative to chow controls (Fig. 4*A,B*), which corresponded to a ∼35% reduction in home-cage activity levels as measured by RAD (Fig. 4*C*,*D*). This demonstrates that RAD is capable of detecting and quantifying within-mouse changes in activity levels over time.

## Discussion

Here, we present a PIR-based device, RAD, which can be used to monitor activity in rodent home cages. To investigate its utility, we completed three sets of validation experiments. In the first dataset, we used simultaneous video monitoring and determined that the PIR is useful for measuring changes in locomotion over minutes, but not on a second-by-second time-scale. In our second dataset, we measured continuous activity data from 40 cages for 8 d without any investigator interference, demonstrating its utility in circadian monitoring. In our third experiment, data collection was completed over nine weeks to track the activity levels of mice fed either a HFD diet or a control diet. Apart from battery replacement every two to three weeks and changing food and water weekly, this experiment was also accomplished with no experimenter intervention. These experiments demonstrate the utility of a simple PIR-based logging device, RAD, in high-throughput studies of rodent activity. We have released this device as open-source, and have made all code and design files available as supplements to this paper, and online.

### Comparison with current technologies

Commercial solutions have started emerging for monitoring home-cage activity (Iannello, 2019). While technically excellent, these devices require specialized equipment and caging and thus may not be feasible for many research labs. We aimed to create an open-source, cost-effective activity monitor that could be placed in existing home cages and not require a large equipment expense.

Other open source methods to track rodent activity have also previously been used for arena-based settings (Gibbs and Crosbie-Watson, 2017; Kumbol et al., 2018) and home-cage monitoring (Brown et al., 2016; Genewsky et al., 2017). Kumbol et al. (2018) built a device comparable in cost to RAD that can be assembled in a lab and used to monitor rodent movement via an array of infrared beams. Other cost-effective open-source methods have employed video tracking to track activity in an arena (Lopes et al., 2015; Gibbs and Crosbie-Watson, 2017). Home-cage monitoring methods have used microwaves (Genewsky et al., 2017), or PIR sensors (Brown et al., 2016). Brown et al. (2016) used PIR sensors that are similar to those used in RAD to construct a device for tracking mouse movement in home cages. Genewsky et al. (2017) used a different sensing technology, microwave radiation, and published a device that utilizes straightforward circuitry, simple assembly, and inexpensive components to monitor mouse home-cage activity. RAD complements these prior devices and offers a simple battery powered home-cage activity monitor that is optimized for high-throughput experiments with minimal human intervention.

### Device limitations

While RAD is useful for tracking rodent activity, it has limitations and may not be ideal for all applications. One limitation of the RAD is the power source. The battery pack must be charged every two to three weeks, which could limit use of the RAD for long-term monitoring. We envision two potential solutions for this issue. First, our design was optimized for ease of construction, and not for low power consumption. In future revisions, we may be able to optimize the hardware and microcontroller code to reduce power usage. Based on the use of PIRs in consumer-grade home security products, we believe that a battery life of one year for such a device is feasible. In addition, RAD can be powered indefinitely by cable, eliminating the need for a battery. While this introduces the additional challenge of cable management, in certain installations this may be preferred over changing batteries.

An additional limitation lies in the use of a PIR sensor instead of a camera, although we have demonstrated here that such sensors reliably report a proxy of ambulatory activity, they cannot provide information about other rodent movements, such as rearing or grooming. Therefore, PIR sensors are not well suited to detecting and monitoring behavior other than locomotion. Small movements of the front or hind limbs, breathing, or changes in posture are unlikely to be recorded by RAD. The smallest movements able to be reliably recorded are short bouts of locomotion where the mouse is moving at >1 cm/s.

A third limitation of the device is the positioning within the cage. As we are only using one PIR sensor, the cage volume may include areas that are outside the field of view of the sensor, for instance, behind the food hopper (Fig. 1*E*). The PIR sensor position may be moved to reduce this possibility, for instance by positioning the sensor through a hole in the cage, or attached to a side-wall. While RAD measures only a subset of rodent activity, our validation experiments demonstrate that it correlates well with average locomotion speed. In addition, as long as the position is held consistent between devices RAD can be used for quantitative comparisons both within and between mice.

A fourth limitation is the inability of the RAD to monitor group housed animals. As the RAD cannot distinguish between animals, it is best used with singly housed rodents. This limitation also occurs with many other methods for measuring rodent activity, although recent developments in video analysis are starting to track activity of multiple mice in group housed settings (Giancardo et al., 2013; Matsumoto et al., 2013; de Chaumont et al., 2018). While promising, such methods are computationally expensive and difficult to deploy in rodent home cages, making them more suitable for smaller-scale installations in specialized arenas.

A final limitation is that the RAD device was made to be compatible with a specific cage model that is used in our animal facility (Allentown NexGen caging). It is possible that RAD may perform worse in other caging, a possibility that will need to be evaluated by other users of the device. We do not anticipate this being the case if the caging allows for adequate line-of-sight between the RAD and the mouse. While we optimized our design for this caging, we made all design files open-source so that others can modify the design to fit in their caging.

### Potential future improvements

Several improvements may be made to future versions of this device. First, the device data-logging platform can be expanded to include additional sensors. For instance, temperature and humidity are vital aspects of rodent living environment, yet current standards of care usually include whole-room (or whole-vivarium) measurements. In-cage measurements of temperature and humidity would provide a far more detailed picture of the variance experienced across individual cages and could easily be added to RAD with an inexpensive (∼$10) modification. Similarly, additional PIR sensors could be added to reduce “blind spots” and cover more of the floor area of a cage. The open-source nature of the RAD makes such modifications relatively easy to implement.

Collecting the data with the SD cards used in our 20-cage study (Fig. 4) was time intensive and would likely preclude scalability of RAD to higher numbers of cages. This hurdle might be overcome by modifying RAD to transmit data wirelessly to a cloud server. Wireless data transfer would simplify scaling RADs to higher numbers of cages, enable real-time data viewing, and facilitate multi-site studies, where RAD devices could be placed in multiple research facilities. Again, due to the open-source nature of the RAD, adding a wireless radio for data transmission is relatively easy to implement.

## Conclusion

This work provides a resource for investigators studying physical activity in rodent models. The open-source availability and scalability attributes of RAD could facilitate reproducibility of studies across research centers. Continued improvement of methods for monitoring physical activity in animal models may ultimately lead to more effective interventions for increasing physical activity and improving health.

## Synthesis

Reviewing Editor: Christophe Bernard, INSERM & Institut de Neurosciences des Systèmes Decisions are customarily a result of the Reviewing Editor and the peer reviewers coming together and discussing their recommendations until a consensus is reached. When revisions are invited, a fact-based synthesis statement explaining their decision and outlining what is needed to prepare a revision will be listed below. The following reviewer(s) agreed to reveal their identity: Dirk Isbrandt, Lisa Genzel.

We all agree that the ms presents a device useful the community.

Two issues need to be addressed:

1) Could you comment on whether the system needs a hole in the cage wall in order to monitor from the side or is the sensitivity high enough to go through the cage material? If so, it would be helpful to have the system tested on different cage materials (e.g., Tecniplast is a major vendor in Europe) to evaluate the compatibility with their sensor?

2)You explain that resting, grooming, rearing, sniffing and digging were not seen as activity by the device. This restricts the usability of the device to monitor true activity, circadian rhythms and sleep. In particular, you state that circadian rhythms can be measured, which is not really the case: it is circadian rhythms during active wake, not quiet rest. These caveats should be mentioned. Could the authors describe the smallest movement that can be detected?

## Author Response

We thank the reviewers for noting that our device is a useful contribution to the study of physical activity, and acknowledge the two concerns they brought up. We believe we have addressed both in this revised manuscript. Specific responses below:

1. Could you comment on whether the system needs a hole in the cage wall in order to monitor from the side or is the sensitivity high enough to go through the cage material? If so, it would be helpful to have the system tested on different cage materials (e.g., Tecniplast is a major vendor in Europe) to evaluate the compatibility with their sensor?

Response: We apologize for not being clear on this point, as it is relevant to the overall function of the device regardless of its placement. Because of the mechanism of passive infrared sensing, detection requires a clear “line of sight” to the animal. This means that it cannot detect mice through the plastic wall of a cage, although it can be placed behind metal gratings such as a wire food rack that many cages use. While we considered other types of motion sensors that can detect through the wall of a cage, they introduce the potential for false positives, as they can detect activity in the room or in other cages. As PIR sensors do not detect signals through plastic or glass, they can be used with multiple cages situated directly next to one another, such as on a vivarium rack, which was our primary use case. We discuss this point under Method of sensing, and have now clarified in lines 204 - 207 that if a side sensing position is used, RAD should be mounted behind a barrier, such as a wire-grate. If a within-cage side-mount is desired, modifications to the 3D housing file we designed can be implemented such that the body of the device except the PIR sensor itself is protected from the animal.

2. You explain that resting, grooming, rearing, sniffing and digging were not seen as activity by the device. This restricts the usability of the device to monitor true activity, circadian rhythms and sleep. In particular, you state that circadian rhythms can be measured, which is not really the case: it is circadian rhythms during active wake, not quiet rest. These caveats should be mentioned. Could the authors describe the smallest movement that can be detected?

Response: We thank the reviewer for making the important distinction that this device would only pick up circadian patterns as defined by active wake states, and now include this point in the manuscript (line 222). We also expanded the discussion of the information able to be reliably recorded by this device (lines 288-290), and clarified that the slowest movement that can be detected is ~1cm/s of locomotion (ie: not grooming in place).
